# The Milk Committee of the Buffalo Fresh Air Mission Unfolds Its Plans and Objects—Names Its Stations and Gives Information as to How Pure Milk Is Available

**Published:** 1909-12

**Authors:** A. Conger Goodyear, Belle R. Laverack, Henry Vom Berge, N. G. Russell, J. S. Otto, J. A. Ragone

**Affiliations:** Buffalo, N. Y.; Buffalo, N. Y.; Buffalo, N. Y.; Buffalo, N. Y.; Buffalo, N. Y.; Buffalo, N. Y.


### CORRESPONDENCE.

**The Milk Committee of the Buffalo Fresh Air Mission Unfolds Its Plans and Objects—Names Its Stations and Gives Information as to How Pure Milk is Available.**

*Editor Buffalo Medical Journal:*

*Sir:* The Milk Committee of the Buffalo Fresh Air Mission takes this occasion to present to the medical profession a statement of its aims and the method of procedure in the conduct of the milk stations established by it, believing that advantage would be more generally taken of them if the scope of its endeavors were known. Since July, first a central station has been maintained at Welcome Hall and distributing stations at Welcome Hall, Watson House and the District Nursing Association. At each of these stations a physician, assisted by a trained nurse, has been in attendance. In so far as the funds at the disposal of the Committee will permit, additional stations will be opened in localities where the need is demonstrated. The objects striven for are: the encouragement of breast feeding, the education of mothers in infant feed—and hygiene and the providing of pure milk in individual "feedings" for such infants as require artificial food.

To attain these objects the procedure in the conduct of the stations has been as follows: no mother applying directly to the stations can get the milk without first seeing the committee's physician and making it clear that she is unable to nurse her baby. The physician then examines the child, writes a suitable prescription and requests the mother to bring her baby weekly to be weighed and to send daily for the milk. Both oral and printed instructions in hygiene and the care of the milk are given to the mother. These are followed by a visit to the home by the nurse who makes sure that the instructions are understood and carried out. When cases are referred to the stations by family physicians the prescriptions sent by them are filled and visits to the homes by the nurses are made only upon request. The price of the milk and the available formulæ are shown on the physician's order blank enclosed herewith. Mothers who are able are expected to pay the full price of the milk. To the extent of its means the committee will bear the expense of supplying milk to those unable to pay or able to pay only a part of the price. The committee maintains a careful supervision of the production of the milk, which is procured from tuberculin tested cows. A check is kept on the quality of the milk by the city Department of Health, which makes two bacterial counts a week.

A. Conger Goodyear, Chairman.
Belle R. Laverack.
Henry vom Berge.
N. G. Russell, M. D.
J. S. Otto, M. D.
J. A. Ragone, M. D.

Buffalo, N. Y., November 1, 1909.

